# Lateral Spinal Curvature—Urethroplasty

**Published:** 1915-05-08

**Authors:** 


### MODERN METHODS SERIES.

### Lateral Spinal Curvature—Urethroplasty.

Any pronounced deformity leads not only to physical discomfort but to mental suffering. Formerly incurable deformities led indirectly to insanity far more than is possible at the present day, when the general outlook in regard to such matters is more humane. The invalid who withdraws from the society of his fellows, owing to a consciousness of physical malformation, quickly becomes morbid in thought, and is likely sooner or later to harbour a resentment against those more fortunate than himself, and to mature an antagonism which may subsequently develop into serious delusions of a persecutory kind. Obviously all efforts to relieve deformities must be limited by structural changes underlying them, and the greatest relief can only be given where the abnormal feature is chiefly due to some neuromuscular abnormality. Scoliosis or lateral curvature of the spine is a common deformity seen frequently in early years which is likely to change the whole course of life where not attended to, but which fortunately yields to proper treatment if taken in time. In this connection Mr. Paul Bernard Roth, M.B., F.R.C.S., senior orthopædic clinical assistant at the London Hospital, has lately been continuing the investigations carried out by his father and grandfather. In various reports which he has recently published of his system of remedying this deformity Mr. Roth has been careful to distinguish how far the results obtained must necessarily be limited by structural changes; but, in spite of this limitation, he has been able to record numerous examples showing that in many instances the main part of the curving is postural and so amenable to relief.

### The "Best Possible Position."

Accepting the view that definite structural change, in the ribs for example, can never be eliminated, he nevertheless holds that a "best possible position" can be obtained that does away with a great deal of the deformity, and that the first endeavour of the surgeon should be to change the customary position of exaggerated curvature which will usually be found to have become habitual, into this "best possible position," subsequently endeavouring by means of suitable exercises to convert this latter into a natural habit of body. Mr. Roth lays special stress on clothing which, although fitting comfortably when the habitual humped position is maintained, will nearly always be found much too tight when the "best possible position" aimed at is secured. It is accepted as an axiom of the treatment of lateral spinal curvature by exercises that no good will be done if there is any restriction from clothing. The exercises to maintain and fix the "best possible position" are carried out daily, being graduated according to the requirement of individual patients. For them a certain amount of apparatus is required, including a special ladder for extension. A further important feature of this modern method of relieving lateral curvature is the persistent re-education of the muscular sense, with the object of getting the patient to become accustomed to the new habit, and to hold it in mind constantly as it were; whilst at the same time he is advised to obtain plenty of fresh air and reasonable exercise, with a good nourishing diet and long nights. Various home exercises are given to be done twice daily for some months, and every encouragement is afforded to the patient to persist in the maintenance of the "best position" obtained by the surgeon's efforts.

### Urethroplasty.

For many years plastic operations on the surface of the body have been a matter of ordinary routine surgical practice, and skin-grafting, for example, is one of those comparatively simple but exceedingly useful processes the student is taught early in his career. But although experimental work has shown that it is possible to transfer many tissues other than skin from one part to another, or from one animal to another, very little progress has been made in the adaptation of the lessons thus learnt to the relief of human suffering. Even the remarkable researches of Carrel, conclusive as are the results in grafting to which they have led, have not so far greatly aided the surgeon, although it can scarcely be doubted that they will be productive of vast assistance to the plastic surgery of the future. Some presage of the triumphs which will ultimately be scored in this direction is to be found in the report of an interesting example of urethroplasty just made to the *British Medical Journal* by Mr. Noel Braham, F.R.C.S., of Southampton. This was a case in which rupture and subsequent obliteration of the perineal portion of the urethra had occurred in a boy as the result of a fall astride some railings. It was found impossible to reconstitute the natural urethra, which had suffered from sloughing consequent on urinary extravasation, and for six months urine was entirely passed through a suprapubic opening. Subsequently Mr. Braham decided to follow the example of Lexer and Streissler, both of whom have lately reported successful use of the appendix in urethral grafting. Using the boy's vermiform appendix—removed for the purpose—he prepared about an inch thereof, and threading it over a rubber catheter, joined the cut ends to the patent orifices of the divided urethra. A brilliant success was obtained, the patient's natural functions being thoroughly restored. Such a satisfactory solution of an exceedingly difficult problem clearly opens the way to a mending of various other important tubes, such as the vas deferens or ureter. It would appear that an essential element to success is the transplantation of tissues from one part to another of the same individual, rather than from one individual to another—a supposition that, as pointed out by Mr. Braham, is supported by Axhausen's experiments.

